# Pharmacokinetics of carfilzomib in patients with advanced malignancies and varying degrees of hepatic impairment: an open-label, single-arm, phase 1 study

**DOI:** 10.1186/s40164-017-0086-1

**Published:** 2017-10-03

**Authors:** Jennifer Brown, Ruth Plummer, Todd M. Bauer, Stephen Anthony, John Sarantopoulos, Filip De Vos, Mike White, Marco Schupp, Ying Ou, Ulka Vaishampayan

**Affiliations:** 1 Beatson West of Scotland Cancer Centre, 1053 Great Western Rd, Glasgow, Scotland G12 0YN UK; 2 Sir Bobby Robson Cancer Trials Research Centre, Freeman Hospital, Newcastle, England UK; 3 Sarah Cannon Research Institute/Tennessee Oncology, Nashville, TN USA; 4 Arizona Oncology, Sedona, AZ USA; 5 Institute for Drug Development, Cancer Therapy and Research Center, University of Texas Health Science Center San Antonio, San Antonio, TX USA; 6 Department of Medical Oncology, University Medical Center Utrecht, Utrecht, Netherlands; 7 Department of Biostatistics, Amgen Inc., South San Francisco, CA USA; 8 Department of International Development, Amgen (Europe) GmbH, Zug, Switzerland; 9 Department of Clinical Pharmacology, Modeling, and Simulation, Amgen Inc., South San Francisco, CA USA; 10 Department of Oncology, Barbara Ann Karmanos Cancer Institute, Detroit, MI USA

**Keywords:** Carfilzomib, Pharmacokinetics, Advanced malignancy, Hepatic impairment, Oncology, Proteasome inhibitor

## Abstract

**Background:**

Carfilzomib is approved in the United States and Europe for treatment of relapsed or refractory multiple myeloma (MM). This study evaluated pharmacokinetics (PK) and safety of carfilzomib in patients with relapsed or progressive advanced malignancies and varying degrees of impaired hepatic function.

**Methods:**

Patients with normal hepatic function (normal) or hepatic impairment (mild, moderate, or severe) received carfilzomib infusion in 28-day cycles. The primary objective was to assess the influence of hepatic impairment on carfilzomib PK following 27 and 56 mg/m^2^ doses.

**Results:**

The majority of patients enrolled in this study had solid tumors (n = 44) vs. MM (n = 2) since patients with multiple myeloma do not tend to have severe hepatic impairment in the same way as patients with solid tumors. A total of 11 normal and 17 mild, 14 moderate, and 4 severe hepatic impairment patients were enrolled. Compared with patients with normal hepatic function, patients with mild and moderate hepatic impairment had 44 and 26% higher carfilzomib AUC_0–last_, respectively (27 mg/m^2^ dose); increases at the 56 mg/m^2^ dose were 45 and 21%, respectively. Considerable PK variability (% coefficient of variation in AUC ≤100%) was discerned and no consistent trend of increasing exposure resulting from increasing hepatic impairment severity (moderate vs. mild) was seen. The observed adverse event (AE) profile in patients of mostly solid tumors was consistent with the known safety profile of carfilzomib, with the exception of an increased frequency of AEs consistent with hepatic function abnormalities.

**Conclusions:**

In this population of primarily advanced solid tumor patients, patients with mild and moderate hepatic impairment had approximately 20–50% higher carfilzomib AUC vs. normal hepatic function patients. These increases are unlikely to be clinically significant, in light of the intrinsic PK variability and exposure–response relationship of carfilzomib.

*Trial registration* http://clinicaltrials.gov NCT01949545; date of registration: September 6, 2013

**Electronic supplementary material:**

The online version of this article (doi:10.1186/s40164-017-0086-1) contains supplementary material, which is available to authorized users.

## Background

Carfilzomib is an irreversible, proteasome inhibitor approved in the United States of America (USA) and Europe for the treatment of relapsed or refractory multiple myeloma in combination with lenalidomide + dexamethasone or with dexamethasone alone [1]. Carfilzomib is administered by intravenous (IV) infusion and has been shown to be rapidly and extensively metabolized, primarily via extrahepatic pathways, principally peptidase cleavage and epoxide hydrolysis in human and animal studies [2, 3]. Wang and colleagues found that, following IV administration (in patients with solid tumors), plasma concentrations of carfilzomib decline rapidly in a biphasic manner with most of the drug eliminated from the plasma compartment within 30 min. The estimated clearance is high and exceeds hepatic blood flow. The predominant metabolites (M14, M15, and M16) in plasma are formed rapidly, following carfilzomib administration by peptidase cleavage and epoxide hydrolysis. These metabolites have no known biological activity in humans and are inactive as proteasome inhibitors. Cytochrome P450-mediated mechanisms play a minimal role in the overall metabolism of carfilzomib [2].

Within the two phase 3 studies, PX-171-0009 (ASPIRE) and 2011-003 (ENDEAVOR), the eligibility criteria allowed for the enrollment of multiple myeloma patients with baseline mild hepatic impairment (alanine aminotransferase [ALT]/aspartate aminotransferase (AST) >upper limit of normal (ULN) or total bilirubin >ULN) without a starting dose adjustment [4, 5]. Data from these phase 3 studies and other phase 1 and 2 studies were included a population pharmacokinetic (PK) analysis, which indicated no statistically significant differences in PK parameters between subjects with baseline mild hepatic impairment (N = 143) and subjects with baseline normal hepatic function (N = 474) [6]. Of note, patients with multiple myeloma do not tend to have severe hepatic impairment in the same way as patients with solid tumors [7, 8]. Since patients with ALT/AST ≥3 × ULN and bilirubin ≥2 × ULN were excluded from the carfilzomib clinical trials, this dedicated hepatic impairment study evaluated the PK and safety of carfilzomib in patients with relapsed or progressive advanced malignancies and varying degrees of impaired hepatic function.

## Methods

### Patients

Patients aged ≥18 years with relapsed or progressive advanced malignancies (hematologic or solid tumor) were eligible for enrollment. Patients must have received ≥2 prior treatment regimens or not have available other treatments considered standard of care. Other inclusion criteria included Eastern Cooperative Oncology Group performance status (ECOG-PS) 0–2 and adequate renal function (creatinine clearance ≥30 mL/min), absolute neutrophil count (ANC) ≥1.0 × 10^9^/L, hemoglobin ≥8 g/dL, and platelet count ≥50 × 10^9^/L (or ≥30 × 10^9^/L, if bone marrow disease involvement was >50%) documented within 21 days prior to enrollment. Exclusion criteria included myocardial infarction within 6 months of enrollment; as well as current or recent congestive heart failure (New York Heart Association class III or IV), symptomatic ischemia, or uncontrolled conduction abnormalities. Patients were excluded if they had known HIV or hepatitis B or C virus infection (except chronic or cleared hepatitis infection with stable liver function tests); neurotoxicity of grade ≥2 severity; symptomatic brain metastasis or central nervous system disease; active infection requiring systemic antibiotics, antiviral (except against hepatitis B), or antifungal treatment within 2 weeks of enrollment; uncontrolled hypertension or diabetes within 2 weeks of enrollment; any investigational product, systemic antineoplastic therapy, or focal radiotherapy within 1 week, immunotherapy within 3 weeks, or major surgery within 3 weeks of enrollment.

Hepatic impairment was required to be stable with no acute worsening of liver function within 1 month prior to enrollment. Level of impairment was assessed according to National Cancer Institute Organ Dysfunction Working Group (NCI-ODWG) schema with patients categorized by hepatic function: cohort 1 (normal) had bilirubin and AST levels ≤ULN; cohort 2 (mild) had bilirubin 1.0–1.5 × ULN, or AST > ULN with bilirubin ≤ULN; and cohort 3 (moderate) had 1.5–3.0 × ULN with any AST. The original protocol included a fourth cohort with severe hepatic impairment (bilirubin >3 × ULN and any AST), but was discontinued per protocol amendment due to enrollment challenges and the lack of demonstrable efficacy with carfilzomib monotherapy in this population of mostly advanced solid tumors. These patients were not included in the PK-evaluable population, but were included in the safety population.

### Study design

This was a phase 1, multicenter, open-label, nonrandomized, comparative PK study of carfilzomib in patients with normal hepatic function or mild-to-moderate hepatic impairment. The study was conducted at 12 sites in the USA, United Kingdom, France, and the Netherlands. The primary objective was to assess the influence of baseline hepatic impairment on the area under the concentration–time curve (AUC; from time 0 to time of last concentration measured [AUC_0–last_] and from time 0 extrapolated to infinity [AUC_0–inf_]) of carfilzomib at 27 mg/m^2^ in patients with relapsed or progressive advanced malignancies. Secondary objectives were to compare additional PK parameters of carfilzomib 27 mg/m^2^ between hepatic function groups, including maximum plasma concentration (C_max_), time to maximum concentration (T_max_), clearance (CL), terminal half-life (T_1/2_), volume of distribution (V_d_), and mean residence time (MRT); to compare between patients PK parameters of carfilzomib 56 mg/m^2^; to evaluate PK parameters for major metabolites (metabolites PR-389/M14, PR-413/M15, and PR-519/M16); and to evaluate the safety and tolerability of carfilzomib. Exploratory objectives included evaluation of overall response rate (ORR) and duration of response (DOR).

This study (NCT01949545) was conducted in accordance with International Conference on Harmonisation Good Clinical Practice regulations. The protocol and informed consent document were reviewed and approved by each study center’s Institutional Review Board or Independent Ethics Committee. All patients provided written informed consent prior to undergoing any protocol-specific screening procedures or treatments.

**Fig. 1:**
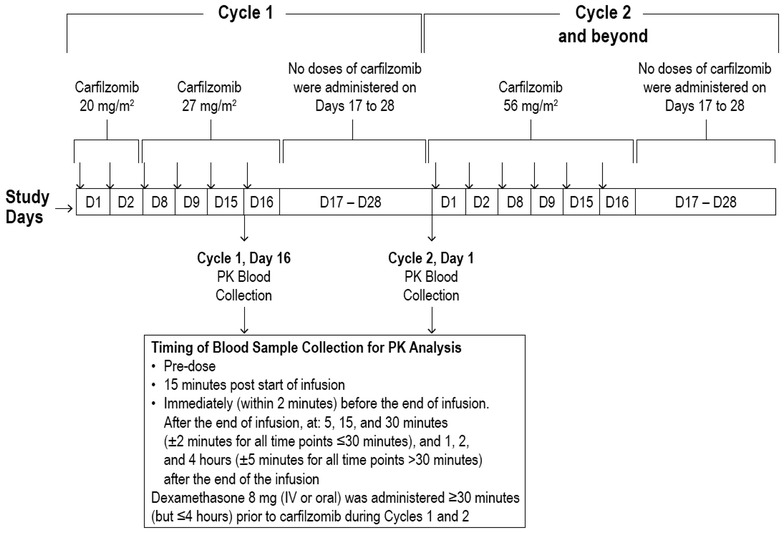
Treatment regimen and PK evaluation protocol. *IV* intravenous, *PK* pharmacokinetic

Patients received carfilzomib as a 30-min IV infusion on days 1, 2, 8, 9, 15, and 16 of 28-day cycles (Fig. 1). All patients were to receive doses of 20 mg/m^2^ on days 1 and 2 of cycle 1. Thereafter, a stepwise dose-escalation regimen was implemented; the carfilzomib dose was increased to 27 mg/m^2^ for days 8, 9, 15, and 16 of cycle 1. For those tolerating the 27 mg/m^2^ dose, carfilzomib was to be increased to 56 mg/m^2^ beginning on day 1 of cycle 2 and throughout subsequent cycles. Dexamethasone 8 mg (IV or oral) was administered at least 30 min (but no more than 4 h) prior to carfilzomib during cycles 1 and 2. If fever, rigors, chills, and/or dyspnea associated with the infusion of carfilzomib were observed post-dose at or after cycle 3 of day 1, dexamethasone continued to be administered pre-dose through cycle 3 and at the investigator’s discretion during subsequent cycles.

To reduce the risk of tumor lysis syndrome (TLS), IV hydration was administered immediately prior to and following carfilzomib dosing during cycle 1 and at the investigator’s discretion in cycle 2 and beyond. Hydration consisted of 250–500 mL IV normal saline or other appropriate IV fluid. Patients were permitted to continue carfilzomib until confirmed progressive disease, unacceptable toxicity, or withdrawal of consent.

Concomitant medications permitted during the trial included: allopurinol (or other approved uric acid-lowering agent) in patients at high risk for TLS, mycostatin or oral fluconazole to prevent oral thrush per investigator’s discretion, anti-emetics and antidiarrheal agents as necessary, and bisphosphonates in multiple myeloma patients per institutional standards of care. Prophylactic antiviral therapy (e.g., valacyclovir) was strongly recommended for patients at increased risk of herpes zoster. Myeloid growth factors were not to be given prophylactically, but were permitted if neutropenia occurred; this is in accordance with the American Society of Clinical Oncology Guidelines [9].

### Assessments

Blood samples were collected to measure PK and plasma concentration of carfilzomib and its major metabolites (PR-389/M14, PR-413/M15, and PR-519/M16). Samples were collected on day 16 of cycle 1 and day 1 of cycle 2 (or on subsequent infusion days when the intended dose levels were administered) before infusion; at 15 min after start of infusion; within 2 min before end of infusion; and at 5, 15, and 30 min (±2 min for each); and at 1, 2, and 4 h (±5 min for each) after end of the infusion (Fig. 1). Safety was assessed throughout the study and included the monitoring of adverse events (AEs) using National Cancer Institute-Common Terminology Criteria for Adverse Events, version 4.03 (NCI-CTCAE v4.03), clinical laboratory evaluations, and vital signs; echocardiograms and physical examinations were also performed. The exact type and frequency of disease or tumor response assessments depended upon tumor type and was assessed by the investigator using the appropriate measure for the underlying malignancy, including Response Evaluation Criteria in Solid Tumours version 1.1.

For multiple myeloma patients, efficacy was assessed on day 1 of each cycle during treatment. For solid tumor patients, efficacy was assessed on day 1 of every other cycle [i.e., on day 1 (±7 days) of cycles 3, 5, 7, 9, etc.] during treatment.

### Statistical analysis

Enrollment of ten PK-evaluable patients per cohort (normal hepatic function, mild hepatic impairment, and moderate hepatic impairment) was planned. Patients who were not considered PK-evaluable were replaced. Descriptive statistics were provided for selected demographics, safety, and PK data, with all statistical summaries and analyses performed in SAS^®^ version 9.1 or higher (SAS Institute Inc., Cary, NC). The primary PK analyses and other summaries of the PK parameters were performed using the PK-evaluable population, defined as patients with adequate carfilzomib plasma concentration vs. time data for the estimation of PK parameters by a non-compartmental analysis. Patients who were not considered PK-evaluable were replaced. Plasma PK parameters of carfilzomib and metabolites were computed in Phoenix WinNonlin^®^ Enterprise.

Individual concentration data for carfilzomib and metabolites were listed and summarized for each nominal time point in accordance with the grouping factors (cohort and dose). PK parameters, including AUC, C_max_, T_1/2_, CL, V_d_, and MRT, were summarized using geometric mean (GeoMean) and geometric coefficient of variation percent (%GeoCV). T_max_ was summarized using median and range. Individual plasma concentration–time profiles were presented on linear–linear and log-linear scales, using the same grouping factors and actual sampling time.

To assess the effect of hepatic impairment on the PK parameters AUC_0–last_ and AUC_0–inf_ of carfilzomib 27 and 56 mg/m^2^, analysis of variance of the ln-transformed plasma PK parameters was performed to calculate the GeoMean ratios, with hepatic impairment as a fixed effect. The point estimates of the GeoMean ratios were calculated by exponentiation of the differences in the least-squares means (LSM), using ln-transformed data, between the hepatic impairment cohorts 2 and 3 (test) and normal hepatic function cohort 1 (reference group). GeoMean ratio 90% confidence intervals (CIs) were transformed similarly by exponentiation of the corresponding 90% CI for the difference between the LSM calculated for the ln-transformed values.

Safety was assessed for patients who received any carfilzomib dose. AEs were mapped to a preferred term and system organ class using MedDRA and were graded using the NCI-CTCAE v4.03. ORR was summarized along with 95% exact binomial CIs by cohorts. Disease response was assessed for those who received ≥1 dose and had a baseline and ≥1 post-baseline assessment.

## Results

### Patients and enrollment

Of 71 patients who were screened for the trial, 46 were enrolled and treated: 11 with normal hepatic function, 17 with mild hepatic impairment, 14 with moderate hepatic impairment, and 4 patients with severe hepatic impairment. Baseline characteristics of the study participants are summarized in Table 1. Median age was 63.5 years (range 37–78 years), with 44% of patients being over age 65. The majority of patients were white (89%), male (61%), and had an ECOG-PS of 1 (61%).

**Table 1:** Patient demographics and baseline disease characteristics (safety population)

Characteristics	Hepatic function				
	Normal (n = 11)	Mild impairment (n = 17)	Moderate impairment (n = 14)	Severe impairment (n = 4)	Total (N = 46)
Sex, n (%)					
Male	9 (82)	9 (53)	9 (64)	1 (25)	28 (61)
Race, n (%)					
Black	0 (0)	1 (5.9)	1 (7)	0 (0)	2 (4)
White	11 (100)	13 (76.5)	13 (93)	4 (100)	41 (89)
Not reported	0 (0)	3 (17.6)	0 (0)	0 (0)	3 (7)
Age, years					
Median	69.0	59.0	62.5	54.5	63.5
Range, min–max	55–78	37–74	47–70	45–71	37–78
≥65 years, n (%)	8 (72.7)	5 (29.4)	6 (42.9)	1 (25.0)	20 (43.5)
ECOG performance status, n (%)					
0 or 1	11 (100)	13 (76)	12 (86)	4 (100)	40 (87)
2	0 (0)	4 (24)	2 (14)	0 (0)	6 (13)
Time from initial diagnosis to informed consent, years					
Median	3.75	2.95	2.34	2.78	2.82
Min, max	0.8, 9.4	0.9, 16.4	0.7, 7.8	1.3, 4.0	0.7, 16.4
Current disease stage, n (%)					
3	0 (0)	0 (0)	3 (21)	1 (25)	4 (9)
4	8 (73)	16 (94)	10 (71)	3 (75)	37 (80)
Unknown	1 (9)	1 (6)	1 (7)	0 (0)	3 (7)
Missing	2 (18)	0 (0)	0 (0)	0 (0)	2 (4)

*ECOG* Eastern Cooperative Oncology Group, *ITT* intent-to-treat, *max* maximum, *min* minimum, *SD* standard deviation

The final study population consisted of patients with mostly solid tumors (n = 44) or multiple myeloma (n = 2) (Additional file 1: Table S1). More than half of the population had colorectal cancer or hepatocellular carcinoma (33 and 24%, respectively). Other types of cancer diagnosed in >1 patient each were pancreatic (7%), breast (4%), small-cell lung (4%), and multiple myeloma (4%). Most patients (80%) had stage 4 disease.

### Carfilzomib PK

The PK-evaluable population included 33 of the 46 enrolled patients: 10 patients with normal hepatic function, 14 patients with mild hepatic impairment, 9 patients with moderate hepatic impairment, and 0 patients with severe hepatic impairment. As noted previously, the original protocol included patients with severe hepatic impairment; however, enrollment was discontinued per protocol amendment due to enrollment challenges and the lack of demonstrable efficacy with carfilzomib monotherapy in this population of mostly advanced solid tumors. None of the enrolled subjects with severe hepatic impairment were PK-evaluable. Although these patients were excluded from PK and efficacy evaluations, they were included in the safety population.

**Fig. 2:**
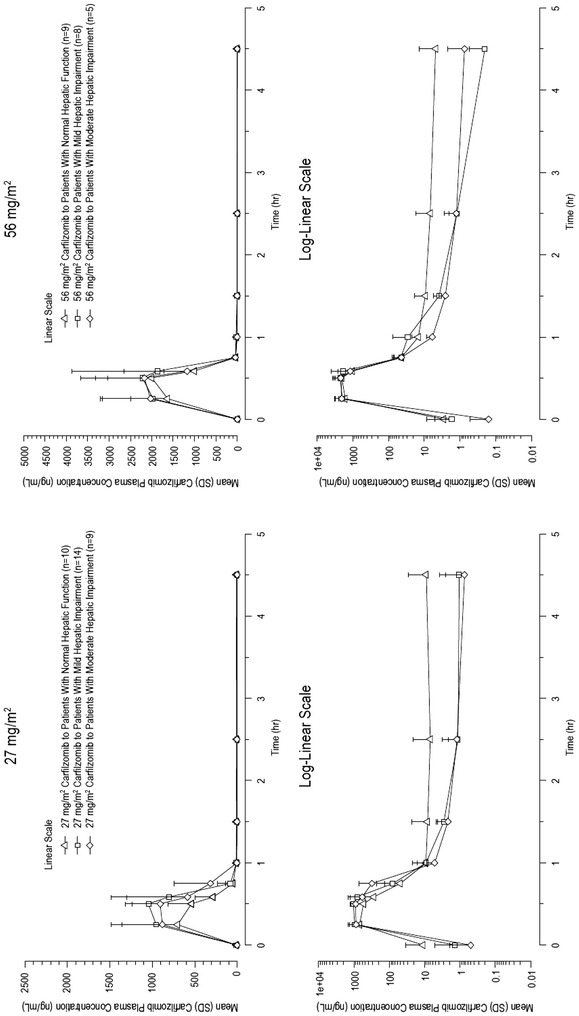
Mean (+SD) plasma concentration–time profiles of carfilzomib following IV administration of carfilzomib (linear and semi-log plots; PK-evaluable population). *IV* intravenous, *PK* pharmacokinetic, *SD* standard deviation

Following IV administration, the plasma concentration of carfilzomib 27 and 56 mg/m^2^ declined rapidly in a bi-phasic and similar manner (Fig. 2). Median T_max_ ranged from 0.29 to 0.48 h (Table 2) with peak concentrations of carfilzomib most often observed 15 min after start or immediately before the end of infusion (Fig. 2). After end of infusion, concentrations of carfilzomib declined rapidly with a mean T_1/2_ of approximately 0.5 to 0.7 h in all patient groups.

**Table 2:** Carfilzomib PK parameters and inferential PK statistics comparing carfilzomib exposure following IV administration of carfilzomib (PK-evaluable population)

PK parameters	27 mg/m^2^ by hepatic function			56 mg/m^2^ by hepatic function		
	Normal (n = 10)	Mild impairment (n = 14)	Moderate impairment (n = 9)	Normal (n = 8)	Mild impairment (n = 8)	Moderate impairment (n = 5)
AUC_0–last_, ng·hr/mL	378 (40.8)	546 (39.2)	477 (33.1)	765 (100.5)	1107 (73.7)	927 (45.8)
AUC_0–inf_, ng·hr/mL	348 (35.4)^a^	529 (40.3)^b^	500 (38.4)^c^	609 (99.6)^d^	1108 (73.7)	929 (46.2)
C_max_, ng/mL	932 (58.4)	1290 (47.5)	1020 (43.7)	1697 (93.7)	2733 (67.0)	2119 (47.9)
T_max_, h	0.292 (0.250–0.500)	0.458 (0.250–0.667)	0.483 (0.233–0.750)	0.300 (0.250–0.583)	0.408 (0.250–0.683)	0.400 (0.250–0.583)
T_1/2_, h	0.469 (22.8)^a^	0.541 (75.9)^b^	0.511 (219.4)^c^	0.508 (54.7)^d^	0.621 (47.7)	0.740 (137.7)

Values presented as geometric mean (geometric CV%); T_max_ median (minimum–maximum is presented). Data in table excludes n = 1 cycle 2 day 1 outlier patient from the normal hepatic function group

*AUC*_*0–inf*_ area under the concentration time curve from time extrapolated to infinity, *AUC*_*0–last*_ area under the concentration time curve from time 0 to last concentration measurement, *C*_*ma*x_ maximum plasma concentration, *T*_*max*_ time to maximum plasma concentration

^a^n = 8

^b^n = 12

^c^n = 7

^d^n = 6

Within each of the PK-evaluable treatment groups, considerable PK variability was observed following IV administration of carfilzomib 27 and 56 mg/m^2^ (Fig. 2). The %CV in AUC_0–last_, AUC_0–inf_, and C_max_ parameters ranged from 33 to 100% (Table 2). Exposure overlapped across the three patient groups.

Of note, high PK variability was observed in AUC_0–last_, AUC_0–inf_, and C_max_ (%CV of ~90 to 100%) for the 56 mg/m^2^ normal hepatic function group due to the presence of an outlier: a patient with a C_max_ of 281 ng/mL at 56 mg/m^2^. This C_max_ was considerably lower than the C_max_ observed at 27 mg/m^2^ for this patient (711 ng/mL), which was unlikely to be biologically plausible. A sensitivity analysis that excluded this patient showed a reduction of %CV for AUC_0–last_, AUC_0–inf_, and C_max_ from 90–100% to 23–39%.

**Fig. 3:**
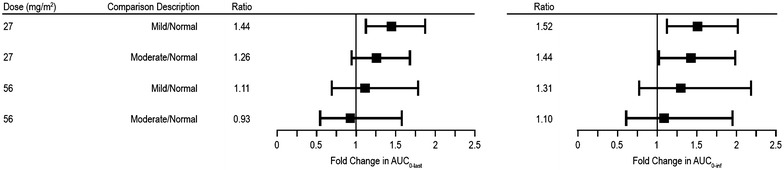
Forest plot of carfilzomib exposure PK parameter ratios and 90% CI following IV administration of carfilzomib *. Horizontal axis shows the fold change in AUC_0–last_ and AUC_0–inf_, relative to the normal hepatic function group. * Excludes 1 outlier patient from normal hepatic function group at the carfilzomib 56 mg/m^2^ dose. *AUC*_*0–inf*_ time 0 extrapolated to infinity, *AUC*_*0–last*_ time 0 to time of last concentration measured, *CI* confidence interval, *IV* intravenous, *PK* pharmacokinetic

A dose-dependent increase in mean AUC and C_max_ of carfilzomib was observed between 27 and 56 mg/m^2^ in all three cohorts (Table 2). There was a trend at both carfilzomib doses toward a higher AUC_0–last_ and AUC_0–inf_, higher C_max_, and slower clearance in patients with baseline hepatic impairment (mild or moderate) compared with normal hepatic function patients. Comparison of total exposure to carfilzomib in patients with mild or moderate hepatic impairment vs. patients with normal hepatic function showed no consistent trend of increasing exposure with increasing severity of hepatic impairment (i.e., patients with moderate hepatic impairment did not have greater exposure compared with mild hepatic impairment patients) (Table 3; Fig. 3).

**Table 3:** Inferential pharmacokinetic statistics comparing carfilzomib exposure following intravenous administration of carfilzomib (PK-evaluable population)

Sampling occasion (dose level)	PK parameters	Geometric LSM by hepatic function		Geometric mean ratios (%) 90% CIs (lower; upper)	p values
		Mild impairment	Normal		
27 mg/m^2^	AUC_0–last_ (ng·hr/mL)	546 (n = 14)	378 (n = 10)	144.43 (111.48; 187.12)	0.02232
	AUC_0–inf_ (ng·hr/mL)	529 (n = 12)	348 (n = 8)	151.84 (113.59; 202.96)	0.02137

		Moderate impairment	Normal		
27 mg/m^2^	AUC_0–last_ (ng·hr/mL)	477 (n = 9)	378 (n = 10)	126.08 (94.59; 168.06)	0.1812
	AUC_0–inf_ (ng·hr/mL)	500 (n = 7)	348 (n = 8)	143.53 (103.28; 199.45)	0.07247

		Mild impairment	Normal		
56 mg/m^2^	AUC_0–last_ (ng·hr/mL)	1107 (n = 8)	765 (n = 8)	144.65 (79.2; 264.2)	0.3020
	AUC_0–inf_ (ng·hr/mL)	1108 (n = 8)	609 (n = 6)	181.90 (96.4; 343.24)	0.1194

		Moderate impairment	Normal		
56 mg/m^2^	AUC_0–last_ (ng·hr/mL)	927 (n = 5)	765 (n = 8)	121.10 (60.93; 240.67)	0.6347
	AUC_0–inf_ (ng·hr/mL)	929 (n = 5)	609 (n = 6)	152.59 (74.87; 310.96)	0.3155

Geometric LSMs are the least squares means from ANOVA presented following back transformation to the original scale. The 90% confidence intervals are presented following back transformation to the original scale

*ANOVA* analysis of variance, *AUC*_*0–last*_ area under the concentration–time curve from time 0 to last concentration measurement, *AUC*_*0–inf*_ area under the concentration–time curve from time 0 extrapolated to infinity, *CI* confidence interval, *LSM* least-squares mean, *PK* pharmacokinetic(s)

Following a 30-min infusion of carfilzomib 27 mg/m^2^, the ratios of the geometric means for patients with mild hepatic impairment vs. normal hepatic function patients for carfilzomib AUC_0–last_ and AUC_0–inf_ were 144 and 152%, respectively; the corresponding ratios of the geometric means for moderately impaired patients vs. normal hepatic function patients were 126 and 144%, respectively. Following a 30-min infusion of carfilzomib 56 mg/m^2^, the ratios of the geometric means for patients with mild hepatic impairment vs. normal hepatic function patients for carfilzomib AUC_0–last_ and AUC_0–inf_ were 145 and 182%, respectively; the corresponding ratios of the geometric means for moderately impaired patients vs. normal hepatic function patients were 121 and 153%, respectively. For 56 mg/m^2^ mild hepatic impairment patients vs. normal hepatic function patients, the 90% CI was 96–343%, consistent with the large variability in AUC_0–last_ and AUC_0–inf_ and the presence of an outlier. Per a sensitivity analysis that excluded the aforementioned outlier, a geometric mean ratio of 111% (cohort 2 vs. cohort 1) and 93% (cohort 3 vs. cohort 1) in overall exposure (AUC_0–last_) was observed with mild or moderate hepatic impairment, respectively, as compared with normal hepatic function. Thus, the totality of data did not support a consistent exposure increase in patients with mild/moderate hepatic impairment compared with patients with normal hepatic function.

### PK of major metabolites

Following administration of carfilzomib 27 or 56 mg/m^2^, the median T_max_ for each of the metabolites was <1 h (0.75–0.99 for PR-389/M14, 0.65–0.80 for PR-413/M15, and 0.48–0.7 h for PR-519/M16). Concentrations declined rapidly (mean T_1/2_ 1.0–1.4, 1.1–1.3, and 0.67–0.79 h, respectively) in all patients, without any clear relationship to the category of hepatic impairment. For each metabolite, dose-dependent increases in mean AUC_0–last_ and C_max_ were observed in all three patient groups. There was no clear trend of difference in M14 AUC and T_1/2_ in patients with impaired hepatic function compared with patients with normal hepatic function. At the 56 mg/m^2^ dose, the mean M14 C_max_ in patients with moderate hepatic impairment was 513 ng/mL compared with 381 ng/mL in normal hepatic function patients. A mean increase of approximately 60–70% for M15 and 60–80% for M16 was observed with respect to AUC_0–last_, AUC_0–inf_, and C_max_ in patients with moderate hepatic impairment vs. normal hepatic function patients.

### Safety

All 46 enrolled patients received ≥1 dose of carfilzomib and were included in the safety evaluation population. Median duration of exposure was 4.2 weeks: 6.0 weeks for normal hepatic function patients, 4.3 weeks for mild hepatic impairment patients, 2.3 weeks for moderate hepatic impairment patients, and 0.8 week for severe hepatic impairment patients. The median number of cycles received was 2.

Treatment-emergent AEs were reported in 98%, while grade ≥3 AEs were reported for 76% of all patients (Table 4). Fifteen patients (33%) experienced grade ≥3 AEs that were considered related to treatment. The incidence of AEs was higher with increasing degree of hepatic impairment, due to the rate of AEs related to hepatic function (increased bilirubin, AST, ALT) which was higher among patients with mild or moderate hepatic impairment vs. patients with normal hepatic function. There were no apparent trends indicating increased incidence of AEs with increasing degree of hepatic impairment for commonly occurring AEs (i.e., fatigue, anemia, diarrhea, nausea, vomiting, decreased appetite).

**Table 4:** Most common treatment-emergent AE by hepatic function (safety population)

AE, n (%)	Hepatic function				
	Normal (n = 11)	Mild impairment (n = 17)	Moderate impairment (n = 14)	Severe impairment (n = 4)	Total (N = 46)
Any AE^a^	10 (90.9)	17 (100.0)	14 (100.0)	4 (100.0)	45 (97.8)
Fatigue	8 (72.7)	7 (41.2)	9 (64.3)	0 (0.0)	24 (52.2)
Blood bilirubin increased	0 (0.0)	1 (5.9)	10 (71.4)	0 (0.0)	11 (23.9)
Anemia	6 (54.5)	10 (58.8)	3 (21.4)	2 (50.0)	21 (45.7)
Peripheral edema	4 (36.4)	2 (11.8)	5 (35.7)	0 (0.0)	11 (23.9)
Diarrhea	4 (36.4)	4 (23.5)	4 (28.6)	1 (25.0)	13 (28.3)
Nausea	3 (27.3)	5 (29.4)	5 (35.7)	0 (0.0)	13 (28.3)
Abdominal pain	0 (0.0)	3 (17.6)	5 (35.7)	1 (25.0)	9 (19.6)
Any grade ≥3 AE	7 (63.6)	12 (70.6)	13 (92.9)	3 (75.0)	35 (76.1)
Grade ≥3 AE reported in >3 patients					
Blood bilirubin increased	0 (0.0)	0 (0.0)	10 (71.4)	0 (0.0)	10 (21.7)
Alanine aminotransferase increased	0 (0.0)	0 (0.0)	4 (28.6)	0 (0.0)	4 (8.7)
Anemia	2 (18.2)	3 (17.6)	1 (7.1)	1 (25.0)	7 (15.2)
Fatigue	2 (18.2)	3 (17.6)	2 (14.3)	0 (0.0)	7 (15.2)
Any grade ≥4 AE	1 (9.1)	4 (23.5)	4 (28.6)	3 (75.0)	12 (26.1)
Treatment-related AE	8 (72.7)	13 (76.5)	12 (85.7)	1 (25.0)	34 (73.9)
Treatment-related grade ≥3 AE	2 (18.2)	5 (29.4)	8 (57.1)	0 (0.0)	15 (32.6)
Treatment-related serious AE	0 (0.0)	3 (17.6)	4 (28.6)	0 (0.0)	7 (15.2)

*AE* adverse event

^a^Any grade AE reported in ≥30% of patients

Serious AEs were reported in 25 patients (54.3%): 27% of normal hepatic function patients, 59% of mild hepatic impairment patients, 57% of moderate hepatic impairment patients, and 100% of severe hepatic impairment. Serious AEs occurring in >1 patient included ascites, sepsis, and acute kidney injury (three patients each); and anemia, lower respiratory tract infection, pneumonia, increased blood bilirubin, and hepatic encephalopathy (two patients each). The elevated incidence of serious AEs in the mild and moderate hepatic impairment cohorts vs. the normal hepatic function cohort was consistent with expected comorbidities associated with the underlying hepatic impairment.

All enrolled patients discontinued treatment (Table 5): AEs resulting in treatment discontinuation in >1 patient included increased blood bilirubin (n = 2 moderate impairment patients) and pneumonitis (one normal hepatic function patient and one moderate hepatic impairment patient). Nine patients died within 30 days after the last dose of carfilzomib. Two deaths, both of which were in the mild hepatic impairment group, were considered related to treatment: gastrointestinal hemorrhage and acute respiratory distress syndrome.

**Table 5:** Treatment discontinuations

Reason for discontinuation, n (%)	Hepatic function				
	Normal (n = 11)	Mild impairment (n = 17)	Moderate impairment (n = 14)	Severe impairment (n = 4)	Total (N = 46)
AEs	1^a^	1	4^a^	0	6
Disease progression	7	13	7	2	29
Investigator decision	0	1	0	0	3
Patient request/consent withdrawn	0	0	2	0	2
Death	1	2	1	2	6

*AE* adverse event

^a^Considered treatment related (pneumonitis in one normal hepatic function patient and one each pneumonitis, infusion reaction, and increased blood bilirubin in the moderate hepatic impairment group)

### Efficacy

The efficacy population consisted of 10 normal hepatic function patients, 15 mild hepatic impairment patients, and 9 moderate hepatic impairment patients. One multiple myeloma patient with normal hepatic function achieved a partial response with a DOR of 3+ months, making the ORR for the overall study population 2.9% (95% CI 0.1–15.3%) (Table 6). An additional 20.6% (7/34) of patients had stable disease (four with normal hepatic function, one mild hepatic impairment, and two moderate hepatic impairment) with duration ranging from 1 to 4.7 months; these patients received a range of 1–6 cycles of carfilzomib.

**Table 6:** Best overall response as determined by investigator (response-evaluable population)

	Hepatic function				
	Normal (n = 10)	Mild impairment (n = 15)	Moderate impairment (n = 9)	Severe impairment (n = 0)	Total (n = 34)
Best overall response—n (%)					
Complete response	0 (0.0)	0 (0.0)	0 (0.0)	0 (0.0)	0 (0.0)
Partial response	1 (10.0)	0 (0.0)	0 (0.0)	0 (0.0)	1 (2.9)
Stable disease	4 (40.0)	1 (6.7)	2 (22.2)	0 (0.0)	7 (20.6)
Progressive disease	5 (50.0)	12 (80.0)	4 (44.4)	0 (0.0)	21 (61.8)
Not evaluable	0 (0.0)	2 (13.3)	3 (33.3)	0 (0.0)	5 (14.7)

Disease response was determined for solid tumor malignancies using Response Evaluation Criteria in Solid Tumors (RECIST)

Disease response was determined for myeloma using the International Myeloma Working Group Uniform Response Criteria (IMWG-URC), except for minimal response which was based on the European Group for Blood and Marrow Transplantation (EBMT) criteria

## Discussion

In this phase 1 trial evaluating the influence of hepatic impairment on PK parameters of carfilzomib in patients with advanced malignancies, we observed no marked differences in exposures (AUC and C_max_) relative to degree of hepatic impairment. As was expected, given the largely extrahepatic mechanisms of carfilzomib metabolism, the effect of hepatic impairment on the PK profile of carfilzomib was not remarkable. There was no consistent trend in carfilzomib exposure for either dose with respect to degrees of hepatic impairment. For example, at the 27 mg/m^2^ dose, patients with moderate hepatic impairment had increased exposures (AUC_0–last_) of 26% (geometric mean ratios), while patients with mild hepatic impairment had increased exposures of 44%, compared with patients with normal hepatic function. The 90% CIs for the geometric mean ratios were large in all instances, given the limited numbers of patients enrolled, consistent with typical hepatic impairment studies [10].

There was considerable PK variability (%CV in AUC up to 100.5%) in each cohort with an overlapping exposure seen among patients with normal, mild, and moderate hepatic impairment. In the normal hepatic function cohort, one outlier contributed to the extremely high variability. This patient had a C_max_ at the 56 mg/m^2^ dose (281 ng/mL) that was approximately ten times lower than the historical data observed at this dose [geometric mean (geometric CV%) of 2079 (44%) ng/mL] [6]. In addition, this low outlier C_max_ value at 56 mg/m^2^ was considerably lower than the C_max_ observed at 27 mg/m^2^ for this same patient (711 ng/mL), which was unlikely to be biologically plausible. While a due diligence follow-up with the clinical site did not identify any reason for the aberrant concentration values observed, an additional sensitivity analysis was conducted. Exclusion of the outlier significantly reduced the CV% for AUC_0–last_, AUC_0–inf_, and C_max_ from 90–100% to 23–39%. Upon excluding the aforementioned outlier, a geometric mean ratio of 111% (cohort 2 vs. cohort 1) and 93% (cohort 3 vs. cohort 1) in overall exposure (AUC_0–last_) was observed with mild or moderate hepatic impairment, respectively, as compared with normal hepatic function. Thus, the totality of data did not support a consistent exposure increase in patients with mild/moderate hepatic impairment compared with patients with normal hepatic function.

With carfilzomib 27 or 56 mg/m^2^, total exposure of the most abundant metabolite, PR-389/M14, was similar across all hepatic function cohorts. For PR-413/M15 and PR-519/M16, a mean increase of approximately 60–80% was observed for M15 and M16 AUC_0–last_, AUC_0–inf_, and C_max_ in patients with moderate hepatic impairment compared with patients with normal hepatic function. As noted previously, these metabolites have no known biologic activity [2, 3].

The effect of hepatic impairment on the PK of carfilzomib was not remarkable, which is consistent with largely extrahepatic mechanisms and a minor role of cytochrome P450-mediated mechanisms in carfilzomib metabolism. In addition, the results from this study are consistent with those of a population PK model that was developed using plasma carfilzomib data from nine clinical trials [6]. Hepatic impairment based on NCI-ODWG, as well as 11 other covariates, were tested in the population PK analysis. The analysis showed no statistically significant differences in PK parameters between patients with mild hepatic impairment (n = 143) vs. normal hepatic function (n = 474). Additionally, an exposure–response analysis including patients from five phase 1b/2 and two phase 3 studies (N = 576) showed that after adjusting for baseline characteristics and prognostic factors, higher exposure (cycle 1 average AUC) of carfilzomib was associated with improved ORR/clinical benefit rate across a dose range of 15–20/56 mg/m^2^, and increasing exposures are not associated with increasing risk of AEs [6]. Thus, a trend toward an increased AUC in patients with mild/moderate hepatic impairment is unlikely to be clinically relevant, in light of intrinsic PK variability and exposure–response relationship shown for carfilzomib.

In the setting of multiple myeloma, for which carfilzomib is an approved therapy, hepatic impairment is not common. Of note, patients with multiple myeloma do not tend to have severe hepatic impairment in the same way as patients with solid tumors [7, 8]. In this study, the majority of cancer patients with baseline hepatic impairment were those with solid tumors. Since hepatic impairment in multiple myeloma patients is rare, it was particularly challenging to find eligible patients with multiple myeloma and moderate or severe hepatic impairment. The hepatotoxic potential of immunomodulatory drugs and proteasome inhibitors is considered low, although the potential for rare cases of drug-induced hepatitis remains. Dosing decisions for the use of novel agents in subjects with multiple myeloma with varying degrees of hepatic impairment are confounded by the typical exclusion of patients with moderate-to-severe hepatic impairment from clinical trials. In this trial of primarily solid tumor patients, while treatment-emergent AEs were reported in all but one patient, there were no apparent trends indicating increased AE incidence with increasing degree of hepatic impairment for commonly occurring AEs, except for the rate of AEs related to hepatic function (increased bilirubin, AST, and ALT) which were, as expected, higher in patients with hepatic impairment vs. those with normal function. The incidence of serious AEs across the three cohorts was also consistent with expected comorbidities associated with the underlying hepatic impairment. All four severe hepatic impairment patients died; two due to progressive disease and two to fatal AEs (one septic shock and one multiple organ failure; both unrelated to treatment).

## Conclusions

In summary, in this population of primarily advanced solid tumor patients, patients with mild hepatic impairment had approximately 44% and patients with moderate hepatic impairment had 21–26% higher carfilzomib exposure (AUC_0–last_) than patients with normal hepatic function. These increases are unlikely to be clinically significant in light of the intrinsic PK variability and exposure–response relationship of carfilzomib. The observed AE profile in this study of mostly solid tumor patients was consistent with the known safety profile of carfilzomib in patients with multiple myeloma, with the exception of increased frequency of AEs consistent with baseline hepatic function abnormalities.

## Additional file

**Additional file 1: Table S1.** Baseline cancer type (safety population).

## Abbreviations

%GeoCV: geometric coefficient of variation percent
AE: adverse event
ALT: alanine aminotransferase
ANC: absolute neutrophil count
AST: aspartate aminotransferase
AUC: area under the concentration–time curve
AUC_0–last_: area under the concentration–time curve from time 0 to time of last concentration measured
AUC_0–inf_: area under the concentration–time curve from time 0 extrapolated to infinity
C_max_: maximum plasma concentration
CI: confidence interval
CL: clearance
DOR: duration of response
ECOG-PS: Eastern Cooperative Oncology Group performance status
GeoMean: geometric mean
NCI-ODWG: National Cancer Institute Organ Dysfunction Working Group
IV: intravenous
LSM: least-squares mean
MRT: mean residence time
NCI-CTCAE: National Cancer Institute-Common Terminology Criteria for Adverse Events
ORR: overall response rate
PK: pharmacokinetic
T_1/2_: terminal half-life
T_max_: time to maximum concentration
TLS: tumor lysis syndrome
ULN: upper limit of normal
USA: United States of America
V_d_: volume of distribution
