# Supplementary material for: Pharmacokinetics of carfilzomib in patients with advanced malignancies and varying degrees of hepatic impairment: an open-label, single-arm, phase 1 study
Source: Exp Hematol Oncol. 2017 Oct 3;6:27. doi: 10.1186/s40164-017-0086-1 (PMC5627448; doi:10.1186/s40164-017-0086-1)
Supplement: Supplementary file 1 — Additional file 1: Table S1. Baseline cancer type (safety population). [file 40164_2017_86_MOESM1_ESM.docx]

**Table S1.** Baseline cancer type (safety population)

|  | **Hepatic function** | | | |  | |
| --- | --- | --- | --- | --- | --- | --- |
| **Baseline cancer type, *n* (%)** | **Normal (n = 11)** | **Mild**  **Impairment (n = 17)** | **Moderate**  **Impairment (n = 14)** | **Severe**  **Impairment (n = 4)** | | **Total (N = 46)** |
| Colorectal | 5 (45.5) | 6 (35.3) | 3 (21.4) | 1 (25.0) | | 15 (32.6) |
| Hepatocellular carcinoma | 0 (0.0) | 1 (5.9) | 8 (57.1) | 2 (50.0) | | 11 (23.9) |
| Pancreatic | 0 (0.0) | 1 (5.9) | 1 (7.1) | 1 (25.0) | | 3 (6.5) |
| Breast | 0 (0.0) | 2 (11.8) | 0 (0.0) | 0 (0.0) | | 2 (4.3) |
| Multiple myeloma | 2 (18.2) | 0 (0.0) | 0 (0.0) | 0 (0.0) | | 2 (4.3) |
| Endometrial/endometroid carcinoma | 0 (0.0) | 1 (5.9) | 1 (7.1) | 0 (0.0) | | 2 (4.3) |
| Gallbladder | 1 (9.1) | 0 (0.0) | 0 (0.0) | 0 (0.0) | | 1 (2.2) |
| Leiomyosarcoma | 0 (0.0) | 1 (5.9) | 0 (0.0) | 0 (0.0) | | 1 (2.2) |
| Lieberkühn adenocarcinoma | 0 (0.0) | 0 (0.0) | 1 (7.1) | 0 (0.0) | | 1 (2.2) |
| Melanoma | 0 (0.0) | 1 (5.9) | 0 (0.0) | 0 (0.0) | | 1 (2.2) |
| Metastatic squamous cell carcinoma | 0 (0.0) | 1 (5.9) | 0 (0.0) | 0 (0.0) | | 1 (2.2) |
| Prostate | 1 (9.1) | 0 (0.0) | 0 (0.0) | 0 (0.0) | | 1 (2.2) |
| Small-cell lung | 0 (0.0) | 2 (11.8)) | 0 (0.0) | 0 (0.0) | | 2 (4.3) |
| Squamous esophageal | 1 (9.1) | 0 (0.0) | 0 (0.0) | 0 (0.0) | | 1 (2.2) |
| Thymic carcinoma | 0 (0.0) | 1 (5.9) | 0 (0.0) | 0 (0.0) | | 1 (2.2) |
| Urothelial carcinoma of bladder | 1 (9.1) | 0 (0.0) | 0 (0.0) | 0 (0.0) | | 1 (2.2) |
|  | | | | | | |
